# Prognostic Biomarkers after Radiotherapy for Nonsmall Cell Lung Cancer Based on Bioinformatics Analysis

**DOI:** 10.1155/2022/6405228

**Published:** 2022-12-15

**Authors:** Kejun Li, Shuang Wang, Na Li, Wenyue Zhao, Ningning He, Jinhan Wang, Kaihua Ji, Manman Zhang, Huijuan Song, Qiang Liu, Liqing Du

**Affiliations:** ^1^Tianjin Key Laboratory of Radiation Medicine and Molecular Nuclear Medicine, Institute of Radiation Medicine, Chinese Academy of Medical Sciences and Peking Union Medical College, Tianjin 300192, China; ^2^Department of Cancer Prevention Center, Tianjin Medical University Cancer Institute and Hospital, National Clinical Research Center for Cancer, Key Laboratory of Cancer Prevention and Therapy, Tianjin Clinical Research Center for Cancer, Tianjin 300060, China

## Abstract

Radiotherapy is one of the main treatment modalities in nonsmall cell lung cancer (NSCLC). However, tumor radiosensitivity is influenced by intrinsic factors like genetic variations and extrinsic factors like tumor microenvironment. Consequently, we hope to develop novel biomarkers, so as to improve the response rate of radiotherapy and overcome resistance to radiotherapy in NSCLC. We investigate the difference genes of primary NSCLC patients before and after radiotherapy in GSE162945 dataset. Gene Ontology (GO), KEGG, Reactome, and GSEA were employed to represent the essential gene and biological function. It was found that most pathway genes clustered in extracellular matrix and ECM-receptor signal pathway. Additionally, TMT-based proteomics was used to survey the differential proteins present in the supernatant of H460 cells before or after irradiation with 2 Gy of *γ*-rays. And then we take the intersection between the proteomics of H460 cell and ECM-receptor signal pathway proteins of GSE162945 datasets. The data revealed that fibronectin 1 (FN1) and thrombin reactive protein 1 (THBS1) were upregulated after radiation in both datasets. Subsequently, survival analyses using the GEPIA web server demonstrated that FN1 and THBS1 had significant prognostic values (Logrank test *P* value < 0.05) for LUAD and LUSC. Our observations from this study suggest that FN1 and THBS1 might have potential to serve as novel biomarkers for predicting NSCLC tumor response to radiotherapy.

## 1. Introduction

Lung cancer is one of the highest incidence and mortality rates of human malignancies worldwide [1]. GLOBOCAN reports that there will be approximately 2.2 million new cases of lung cancer worldwide in 2020, accounting for 11.4% of all malignancies, and approximately 1.8 million deaths, accounting for 18.0% of malignancy-related deaths [2]. Despite the great progress in the treatment of lung cancer, the current prognosis of lung cancer remains poor. Prognostic data based on 61 countries worldwide show that the 5-year survival rate for lung cancer is only 10.0% to 20.0% [3].

Traditionally, lung cancer has been divided into two main histological types: small cell lung cancer (SCLC) and nonsmall cell lung cancer (NSCLC). SCLC is an aggressive form of lung cancer that accounts for 15% of all lung cancer diagnoses, and it is a high-grade neuroendocrine tumor, appearing under the microscope as small round blue malignant cells [4]. It clinically differentiates itself from the more prevalent NSCLC by having a rapid doubling time and high growth rate [5], but highly sensitive to radiotherapy [6]. NSCLC can be mainly divided into three types: adenocarcinoma (ADC), squamous cell carcinoma (SqCC), and large cell carcinoma. NSCLC accounts for approximately 85% of all diagnosed lung cancers, causing a large proportion of lung cancer-related deaths.

In fact, radiotherapy, as an active and effective therapeutic modality, has played a central part in the whole process management of lung cancer [7]. Stereotactic body radiotherapy (SBRT) can be safely and effectively used in patients with inoperable early-stage NSCLC, including those with poor underlying lung function [8]. Despite the excellent local control rate of SBRT in patients with early-stage NSCLC (98% at 3 years and 87% at 5 years), many patients developed distant metastases during follow-up after SBRT; moreover, the emergence of radiation resistance effects during long-term radiotherapy greatly limits patient outcomes [9, 10]. For patients with inoperable stage III NSCLC, the results of trials of consolidation chemotherapy combined with radiotherapy showed no significant improvement in tumor control or patient survival. Consequently, we need to develop novel biomarkers, so as to improve the response rate of radiotherapy and overcome resistance to radiotherapy in NSCLC.

The GSE162945 dataset includes data on primary NSCLC before and after radiotherapy and metastatic NSCLC such as esophageal, cervical, and colon cancers. In metastatic NSCLC, metastatic tumors usually have novel mutations in genes compared with the primary tumors, especially metastases of different origins, and the complexity and heterogeneity of gene mutations increase the uncertainty of the data. Therefore, in order to reduce the interference of metastatic NSCLC genes to the overall data, this study analyzed the primary NSCLC in dataset GSE162945, hoping to identify genes and proteins associated with radiotherapy for NSCLC.

## 2. Materials and Methods

### 2.1. Data Matrix

The GEO database (https://www.ncbi.nlm.nih.gov/geo/) was searched by keywords “non-small cell lung cancer” and “radiation therapy,” and the dataset GSE162945 was found. GSE162945, a dataset containing NSCLC and its metastases as well as tissues after 60Gy stereotactic treatment, was selected for subsequent data analysis.

### 2.2. Screening of Differentially Expressed Genes

The original gene expression set obtained from the download was run through the R package DESeq2.0 (1.32.0) to obtain the differential genes, and mRNA with fold change in a comparison >2 or <0.5 and adjusted significance level *P* < 0.05 were considered differentially expressed.

### 2.3. GO, KEGG, and Reactome Analysis

Significantly differential genes were enriched by gene ontology (GO) function and KEGG pathway analysis. To better observe the pathways enriched for gene changes in lung cancer tissues before and after radiation treatment, Reactome pathway enrichment analysis was also used for significantly differential genes, and these analyses were performed using the g:Profiler database (https://biit.cs.ut.ee/gprofiler/gost). The threshold of significance was set at *P* < 0.05 with an enrichment number ≥5.

### 2.4. GSEA Analysis

To identify signaling pathways that differed between lung cancer tissues before and after radiation treatment, we selected an ordered list of genes by the DESeq2.0 (1.32.0) package and performed gene set enrichment analysis (GSEA) using the easyGSEA website (https://tau.cmmt.ubc.ca/eVITTA/), with mode of analysis selected as preranked GSEA. The databases selected were KEGG, Reactome pathway, WikiPathways, and biological process, adjusted for *P* < 0.05, and the results are listed in order of ES value [11].

### 2.5. Cell Culture and Irradiation

Human NSCLC cells H460 were purchased from the American Type Culture Collection (ATCC) cell bank, and all were cultured in 1640 medium (HyClone) containing 10% fetal bovine serum (HAKATA) in a constant temperature incubator containing 5% CO_2_ at 37°C. The cells were passaged when they reached 80% fusion. Cells in logarithmic growth phase were selected for subsequent experiments.

The 137 Cs *γ*-ray irradiation source (Gamma cell-40 137Cs *γ*-ray irradiation source purchased from Atomic Energy of Canada Ltd.) was used for irradiation at a dose rate of 1 Gy/min.

### 2.6. TMT-Based Quantitative Proteomic Analysis

H460 cells were irradiated for 2 Gy to obtain culture supernatants, which were submitted to Kidio for sequencing by protein extraction, protein digestion and iTRAQ/TMT labelling, high pH reverse phase separation, and low pH nano-HPLC-MS/MS analysis. Protein identifications were accepted if they could achieve an FDR less than 1.0% by the Scaffold Local FDR algorithm.

Protein quantification was carried out in those proteins identified in all the samples with unique spectra ≥ 2. Protein relative quantification was based on the ratios of reporter ions, which reflect the relative abundance of peptides. Proteins with fold change in a comparison > 1.2 or <0.83 and unadjusted significance level *P* < 0.05 were considered differentially expressed.

### 2.7. Survival Analysis

The prognostic impact of relevant gene expression was performed using the GEPIA database (http://gepia.cancer-pku.cn/). GEPIA is a newly developed interactive web server for analyzing the RNA sequencing expression data of 9,736 tumors and 8,587 normal samples from the TCGA and the GTEx projects, using a standard processing pipeline.

## 3. Results

### 3.1. Overview of the mRNA Transcriptome of Primary NSCLC Tissues before and after Radiotherapy Treatment

**Figure 1:**
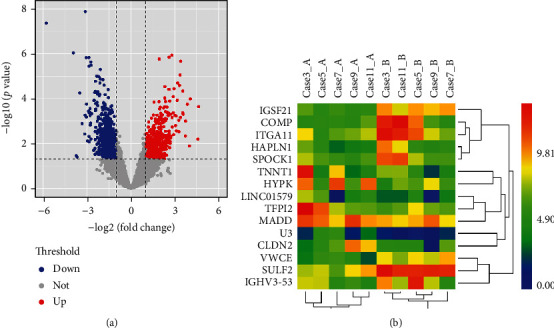
Overview of the differential gene expression between NSCLC and normal tissue. (a) Volcano plot of all proteins with log2 (fc) horizontal coordinate and -log10 (padj) vertical coordinate. (b) Heat map of expression of top 15 DEGs.

The GSE162945 dataset contains transcriptomic data before and after radiotherapy for patients with NSCLC and metastatic lung cancers such as esophageal and colon cancers. To investigate the changes in transcriptomic expression profiles after radiotherapy for primary NSCLC, we performed differential analysis of NSCLC tissues in the GSE162945 dataset before and after radiotherapy by DESeq2.0. In the comparison, genes with fold changes > 2 or <0.5 and an unadjusted significance level of *P* < 0.05 were considered as significantly differentially expressed genes (DEG). After that, 1418 differentially expressed genes were obtained, including 649 genes with upregulated expression and 769 genes with downregulated expression (Figure 1(a)). The differentially aggregated genes were clustered using hierarchical clustering methods, and usually the clustered genes have actual relationships in certain biological processes or in certain metabolic and signaling pathways. In this study, genes with similar accumulation patterns were clustered together, and the top 15 differential genes are shown as a heat map (Figure 1(b)).

### 3.2. Clustering of Biological Processes and Functional Pathway Analysis

**Figure 2:**
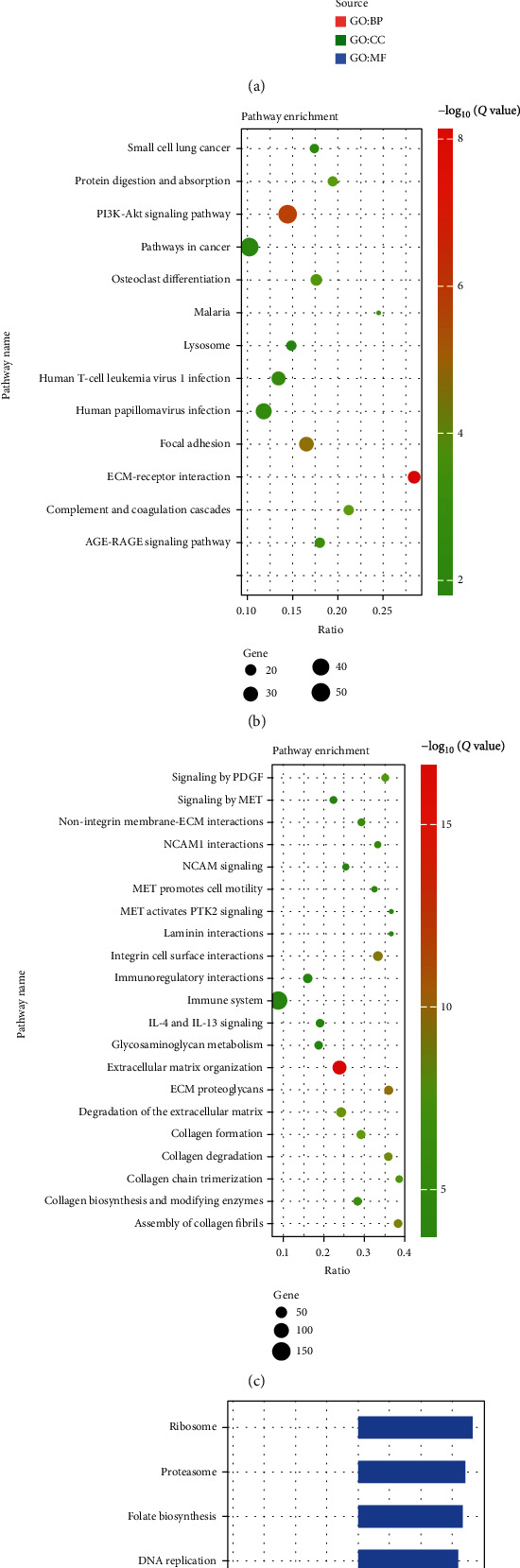
Clustering of biological processes and functional pathway analysis. (a) GO analysis of all differential genes, biological process in red, cellular component in green, and molecular function in blue. (b) KEGG enrichment analysis of all differential genes. (c) Reactome enrichment analysis of all differential genes. (d) GSEA analysis of all genes.

To understand the function and mechanism of these identified DEGs, we used the g:Profiler database to identify GO functional analysis of the upregulated or downregulated differential genes. The top five biologically enriched processes of differentially expressed genes were found to be extracellular structural organization, extracellular matrix organization, external wrapping structural organization, bioadhesion, and cell adhesion (Figure 2(a)); molecularly functionally differential proteins were mainly enriched in extracellular matrix structural components, integrin binding, collagen binding, and extracellular adhesion (Figure 2(a)). In terms of biological processes and molecular functions, they were enriched in extracellular structural components and functions.

KEGG, a classical pathway enrichment pathway, is a strong guide to the function of identified DEGs. KEGG pathway enrichment results showed that differentially expressed genes were mainly in the ECM-receptor interaction, PI3K-Akt signaling pathway, focal adhesion, complement and coagulation cascade, and protein digestion and uptake pathways (Figure 2(b)). To further improve the accuracy of the pathway enrichment, we further analyzed the pathway enrichment using the pathway manually validated Reactome database, showing that DEG was enriched in extracellular matrix organization, ECM proteoglycans, integrin cell surface interactions, assembly of collagen fibrils and other multimeric structures, and collagen degradation pathways (Figure 2(c)). All of these apparently enriched functions and pathways above were focused on extracellular components and extracellular matrix microenvironment.

GO, KEGG, and Reactome databases are all based on overrepresentation analysis, which may appear to discard key genes due to significant differences. In order to observe the overall functional enrichment of genes in lung cancer tissues and corresponding normal lung tissues more comprehensively and to determine the significance of extracellular environment-related gene changes, we analyzed various biofunctional genomes by GSEA. As shown in Figure 2(d), the results showed that the upregulated pathways mainly included ribosome, proteasome, folate biosynthesis, DNA replication, and base excision repair, while the downregulated pathways mainly included AGE-RAGE single conductance pathway, protein degradation and uptake, renin-angiotensin system, ECM-receptor interaction, and glycosaminoglycan biosynthesis. We found that analysis using GSEA would still be enriched in extracellular ECM-receptor interaction pathways.

### 3.3. Identification of FN1 and THBS1 as Promising Predictive Biomarkers after NSCLC Radiotherapy Based on Proteomics and Transcriptomics Analysis

Tumor cell secretions use autocrine and paracrine actions to influence tumor development and prognosis and are also components of the extracellular microenvironment. Therefore, the search for cellular secretions that are significantly altered in the tumor microenvironment after radiotherapy may serve as an indicator of tumor radiotherapy prognosis.

**Figure 3:**
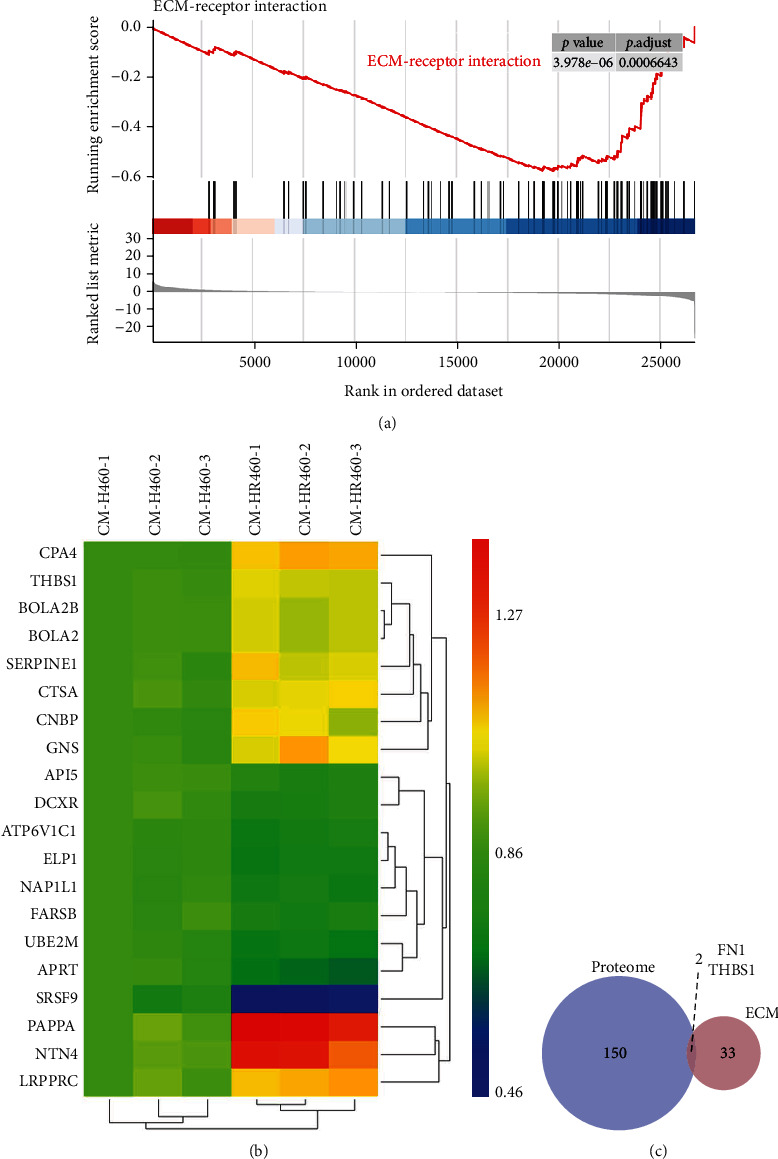
Identification of promising predictive biomarkers after NSCLC radiotherapy. (a) ECM-receptor pathway in GESA. (b) Heat map of expression of top 20 differential proteins in H460 secretions after irradiation. (c) Intersection of proteome sequencing and ECM-receptor pathway.

Therefore, we collected proteins secreted from NSCLC H460 cells cultured in vitro before and after irradiation with 2 Gy *γ*-rays and performed TMT proteomics sequencing analysis. Proteins with fold changes > 1.2 or <0.83 and an unadjusted significance level of *P* < 0.05 were considered as significantly differentially expressed proteins, and 74 proteins with upregulated expression as well as 78 proteins with downregulated expression were obtained. In this study, proteins with similar accumulation patterns were clustered together, and the top 20 differential proteins were shown as a heat map of differentially accumulated proteins (Figure 3(b)).

Subsequently, we combined the 152 differential proteins from proteomics with 35 genes from the ECM-receptor interaction pathway from GSEA analysis and found two genes, FN1 and THBS1, by intersection (Figure 3(c)).

**Figure 4:**
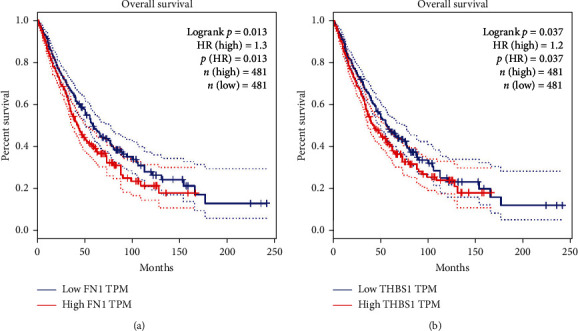
Survival analysis of key genes. Effect of different expression levels of FN1 (a) and THBS1 (b) on survival in lung adenocarcinoma and lung squamous carcinoma.

The relationship between FN1 and THBS1 and the survival rate of patients with lung squamous carcinoma and lung adenocarcinoma were also analyzed using the GEPIA database. It was found that high expression of FN1 and THBS1 was associated with low survival rates in patients with lung adenocarcinoma and lung squamous carcinoma (Figure 4). It showed that these two genes, FN1 and THBS1, were significantly associated with the prognosis of NSCLC, especially with the prognosis of radiotherapy and could potentially be an indicator to assess the efficacy of NSCLC after radiotherapy.

## 4. Discussion

Bioinformatics analysis plays a crucial role in disease research by integrating genome-level data and systematic bioinformatics approaches to facilitate the understanding of disease processes. In this study, we analyzed the differential genes in the GSE162945 database of primary NSCLC tissues before and after radiation treatment and identified a total of 649 genes with an increasing trend and 769 genes with a decreasing trend. Using GO, KEGG, and GSEA analysis, the differential genes were enriched to the ECM-receptor interaction pathway. And later, the differential proteomics secreted by H460 cells before and after irradiation were combined and analyzed, and FN1 and THBS1 were identified as key genes associated with the prognosis of NSCLC radiation therapy.

FN1 is a glycoprotein that exists as a soluble dimer in plasma and as a dimer or multimer on the cell surface and in the extracellular matrix and is involved in cell adhesion and migration processes, including embryogenesis, wound healing, coagulation, host defense, and metastasis. FN1 upregulation and enhanced bridging granule interactions were found to be essential for NSCLC cell aggregation and resistance to apoptosis upon cell detachment from the nest [12]. In patients with NSCLC treated with anlotinib, detection of plasma FN1 levels has potential predictive value for anlotinib efficacy in both the discovery and validation cohorts, suggesting that plasma FN1 levels could be used as a biomarker for anlotinib stratification in NSCLC patients [13]. It indicates that detection of plasma FN1 levels is a clinically feasible assay. Studies on FN1 and radioresistance have focused on patients treated with radiotherapy for head and neck tumors. And among patients with HNSCC (head and neck squamous cell carcinoma) in complete remission and those who failed postoperative radiotherapy, FN1 overexpression plays an important role in the development, prognosis, and radioresistance of HNSCC and is a potential new biomarker for predicting poor prognosis and radioresistance in patients with head and neck squamous cell carcinoma [14]. This study found that FN1 could be used as a potential biomarker for the efficacy and prognosis of radiotherapy in NSCLC. In the present study, only the secretory proteomic data of H460 cells after irradiation were analyzed, which is slightly insufficient. However, Yang et al. analyzed the transcriptome-wide changes in NSCLC A549 cells under radiation and also showed that FN1 was a significant radiation-altered gene [15], providing partial support for our data.

Thrombin-responsive protein 1 (THBS1) is an adhesion glycoprotein that mediates cell-cell and cell-matrix interactions. This protein binds to fibrinogen, fibronectin, laminin, V-type collagen, and integrin *α*-V/*β*-1 to regulate autophagy, senescence, stem cell maintenance, extracellular vesicle function, and metabolic responses to ischemic and genotoxic stress [16]. THBS1 is likewise a clinically feasible assay, and upregulation of THBS1 expression in plasma was detected in osimertinib treatment-resistant NSCLC patients [17]. Our study showed that THBS1 was significantly upregulated in both NSCLC patients after radiotherapy and irradiated H460 cells, and that NSCLC patients with high THBS1 expression generally had a poor survival prognosis, with the potential to predict radioresistance and prognosis in NSCLC patients after radiotherapy.

The tumor immune microenvironment plays an integral role in driving tumor control, tumor progression, and overall survival in patients with NSCLC. The tumor immune microenvironment includes interactions between tumor and immune cells, as well as interactions between immune cells within different tumors. These interactions are highly complex, as almost all types of immune cells can simultaneously infiltrate lung cancer tissue [18]. Conventional radiation therapy approaches can further increase immunosuppression of the tumor microenvironment by destroying many immune cells circulating in the irradiated tumor environment, in addition to direct cytotoxicity to the tumor. Emerging evidence suggests that tumor immunosuppression is a “resilient process” that can be manipulated and transformed back into an immunostimulatory environment to improve patient prognosis [19]. No significant enrichment of immune-related pathways was found in this study, and only a certain number of immune-related genes were enriched in Reactome enrichment analysis, but there was no significant difference. The reason for this may be because the present study focused on the changes in primary NSCLC tissues before and after radiotherapy. When primary and metastatic NSCLC data in the dataset were analyzed together, the most enriched GO enrichment analysis was for immune response genes (6 genes, *P* = 4.67E − 05), and immune response differential genes included TCRs and immunoglobulin superfamily, among others [20]. Compared with primary NSCLC, the tumor immune microenvironment was more significantly altered in metastatic NSCLC after radiotherapy.

In summary, our goal was to identify key proteins produced after radiation therapy for NSCLC through transcriptional and proteomic analysis and to understand the biological functions and signaling pathways in which they are involved. The proteins screened in this study provide new ideas to further explore the impact on the efficacy and prognosis of radiation-treated NSCLC patients. However, the feasibility of these differential proteins as prognostic biomarkers for NSCLC after radiation therapy remains to be further validated.

## Data Availability

The datasets used and/or analyzed during the current study are available from the corresponding author on reasonable request.
